# Physicochemical Characteristics and Antidiabetic Properties of the Polysaccharides from *Pseudostellaria heterophylla*

**DOI:** 10.3390/molecules27123719

**Published:** 2022-06-09

**Authors:** Yingying Liu, Yongjun Kan, Yating Huang, Chang Jiang, Li Zhao, Juan Hu, Wensheng Pang

**Affiliations:** 1 The Second Affiliated Hospital of Fujian University of Traditional Chinese Medicine, Fuzhou 350003, China; liuyy0591@163.com; 2 Institute of Materia, Fujian Academy of Chinese Medical Sciences, Fuzhou 350003, China; kyj@fjtcm.edu.cn (Y.K.); fjjc123@hotmail.com (C.J.); 1181132006@fjtcm.edu.cn (L.Z.); 3 School of Pharmacy, Fujian University of Traditional Chinese Medicine, Fuzhou 350122, China; tzsduotang@163.com

**Keywords:** *Pseudostellaria heterophylla* polysaccharides, physicochemical characteristics, antidiabetic properties

## Abstract

This study aimed to investigate the *Pseudostellaria heterophylla* polysaccharides (PF40) physicochemical and antidiabetic characteristics. The ultraviolet–visible (UV) spectra, Fourier transform infrared radiation (FT-IR) spectra, nuclear magnetic resonance (NMR) spectra, zeta potential, surface characteristics, and conformational and thermal stability properties of PF40 were characterized. X-ray diffraction (XRD) and scanning electron microscopy (SEM), combined with Congo red test, revealed that PF40 powder has mainly existed in amorphous form with triple-helix conformation. The single-molecular structure of PF40 exhibited a multi-branched structure extending from the center to the periphery by scanning probe microscopy (SPM) scanning. The monosaccharide residue of PF40 was an α-pyranoid ring and exhibits good stability below 168 °C. Experimental studies on antidiabetic characteristics found that PF40 could significantly improve STZ-induced intestinal mucosal damage and reduce the apoptosis of villus epithelial cells. PF40 combined with metformin could significantly improve the symptoms of insulin resistance in type 2 diabetes mellitus (T2DM) rats, the molecular mechanism might be through inhibiting the expression of RORγ protein and increasing Foxp3 protein in the jejunum of T2DM rats, and then restoring the STZ-induced imbalance of T helper 17(Th17)/ regulatory T cells (Treg) cells, thereby maintaining intestinal immune homeostasis. Results identified in this study provided important information regarding the structure and antidiabetic characteristics of *Pseudostellaria heterophylla* polysaccharides, which can contribute to the development of *Pseudostellaria heterophylla* polysaccharides for industrial purposes in the future.

## 1. Introduction

T2DM is a metabolic disease with a high incidence that can seriously threaten human health. It is commonly believed that traditional Chinese medicine can delay the progression of diabetes and improve relevant complications [1]. Recently, with the deepening of the research on the mechanism of T2DM treatment with traditional Chinese medicine, attention to the role of intestinal immune balance in the development and maintenance of T2DM is proliferating. In T2DM, chronic intestinal inflammation can promote the occurrence and maintenance of insulin resistance [2,3,4]. The intestinal immune system modulates glucose homeostasis and obesity-associated insulin resistance. Th17/Treg cell balance is crucial for maintaining intestinal immune homeostasis. Insulin resistance can imbalance Th17 cells/Treg cells and inflammation [5,6]. Th17 cells and Tregs cells were functionally considered to be antagonistic to each other, as the Th17 cells are likely to promote immune diseases. At the same time, Tregs can suppress the symptoms of immune disorders that are caused by the Th17 cells [7,8]. Growing evidence suggests that natural polysaccharides are beneficial for maintaining the balance of Th17/Treg cells [9,10,11].

*Pseudostellaria heterophylla* (Miq.) Pax ex Pax et Hoffm. was called Tai-Zi-Shen (TZS). TZS is widely used in the prescription of antidiabetes in China. Polysaccharides have been considered the effective ingredient of TZS. *Pseudostellaria* polysaccharides consist of galactose, glucose, galacturonic acid, arabinose, and rhamnose. It contains non-reducing ends (T-), 1,4-, 1,6- and 1,4,6-linkages, etc., with the main chain consisting of 1,4-linked glucose or galacturonic acid [12].

Our previous studies demonstrated that PF40 could reduce fasting blood glucose and improve insulin resistance in T2DM rats [13]. We further used the radionuclide ^99m^Tc to label the polysaccharides, and the polysaccharides were detected via MRI and PET/CT technology after oral administration in rats. It was found that polysaccharides were mainly enriched in the small intestine [12]. Therefore, we hypothesized that polysaccharides may improve insulin resistance in T2DM rats by regulating intestinal immune homeostasis. However, the mechanism by which PF40 regulates insulin resistance in T2DM rats was unclear. Therefore, based on the extraction, preparation, and structural characterization of PF40, this study was performed to inspect the effect of PF40 on Th17/Treg balance and to examine the medicinal mechanisms of PF40 further.

**Figure 1:**
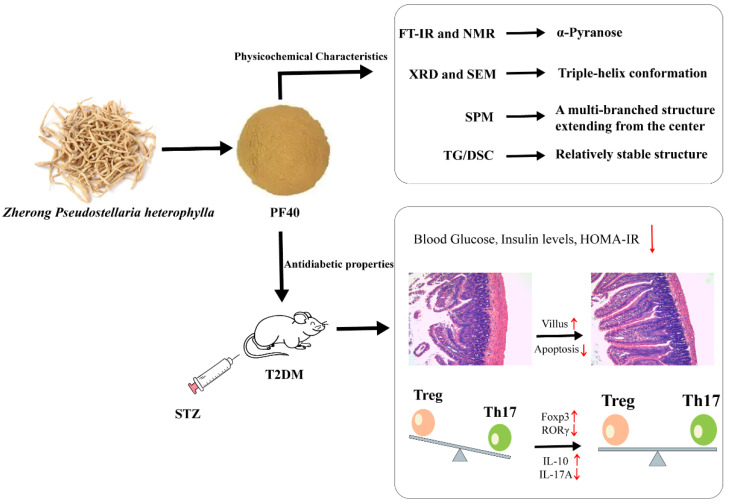
Diagram of the focus of this paper.

In this study, a polysaccharide (PF40) was fractioned from *Pseudostellaria heterophylla* by 40% ethanol precipitation. The physicochemical properties of PF40 were measured, including UV spectra, IR spectra, NMR spectra, and zeta potential. The micropattern and molecular morphology of the extracted polysaccharide were observed using SEM and SPM. The thermal stability properties of PF40 were evaluated using a thermogravimetric analyzer. Congo red experiments were used to explore whether PF40 is triple-helix structured. The antidiabetic properties of PF40 were also investigated to evaluate the potential application of PF40 in the field of polysaccharide drugs. The overview of this paper is shown in Figure 1.

## 2. Results and Discussion

### 2.1. FT-IR Analysis

**Figure 2:**
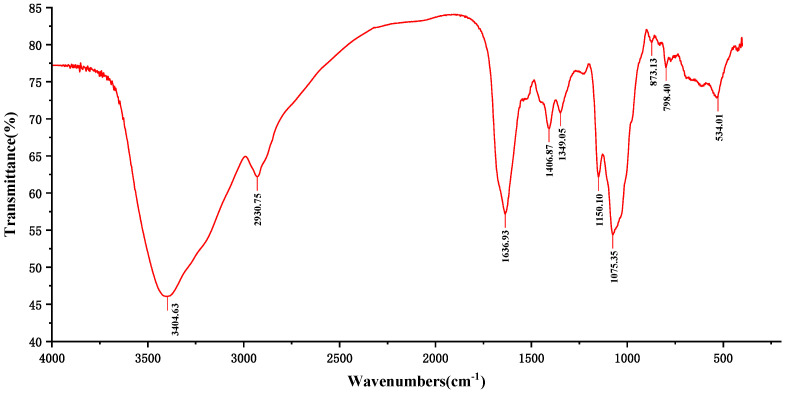
FT-IR spectrum of *Pseudostellaria heterophylla* polysaccharides.

The FT-IR spectrum of PF40 is shown in Figure 2. There were three absorption bands that were caused by the C-H variable angle vibration—namely, the stretching vibration of C-H and O-H at  1406.87  cm^−1^, 2930.75  cm^−1^, and 3404.63 cm^−1^, which were considered characteristic bands of polysaccharide [14,15]. The signal at 1636.93 cm^−1^ correlated with the bending vibration of O-H. We found no bands around 1740 cm^−1^, implying that the PF40 molecule did not possess carbonyl functional groups. In addition, the band around 1074.35 cm^−1^ corresponded to the stretching of the C-O-H or the C-O-C, which might explain the attendance of pyranoid ring conformation in PF40 [16].

### 2.2. NMR Analysis

**Figure 3:**
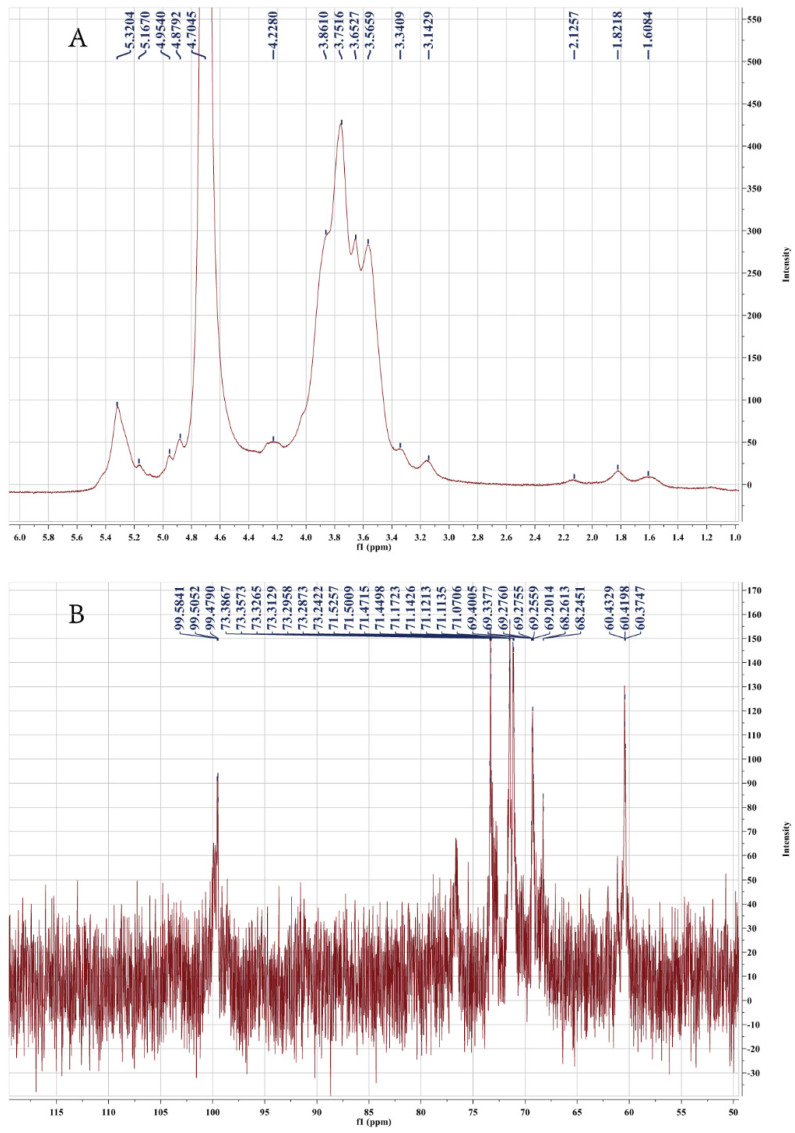
(**A**) ^1^H and (**B**) ^13^C NMR spectra of PF40.

NMR is often used to determine the configuration of polysaccharides. Generally, the signals of the ^1^H NMR spectra in the range of 4.90–5.60 ppm represent anomeric protons of α-anomers, and those in the range of δ4.90–4.20 ppm represent the anomeric protons of β-anomers [17,18]. The ^1^H NMR spectrum of PF40 in D_2_O is shown in Figure 3A. As can be seen, signals appeared at δ5.32 ppm, δ5.17 ppm, and δ4.95 ppm, indicating that PF40 contained α-glycosidic bonds. Furthermore, we found weak signals around δ4.88 ppm and δ4.23 ppm, implying that there was a small percentage of β-glycosidic in PF40. The results of the ^1^H NMR spectrum implied that the main configuration of PF40 was α-configuration.

The anomeric signals in ^13^C NMR were from 95 to 110 ppm, of which 95–101 ppm implied the α-configuration of glucose, and 102–110 ppm implied the type of β-configuration [19]. The ^13^C NMR spectrum of PF40 in D_2_O is shown in Figure 3B. In the ^13^C NMR spectrum, three anomeric carbon signal peaks appeared exactly at 99.5841, 99.5052, and 99.4790 ppm, indicating the existence of three monosaccharide residues in PF40, mainly in the α-configuration, which is compatible with the results of ^1^H NMR. The peaks in the range of 60.37–73.38 ppm are ascribed to C_2_-C_6_ carbon signals. In addition, PF40 has a larger molecular weight and a viscous aqueous solution, making the noise of ^13^C NMR unsatisfactory. Similar results have been reported by other researchers [20,21].

### 2.3. UV Analyses

**Figure 4:**
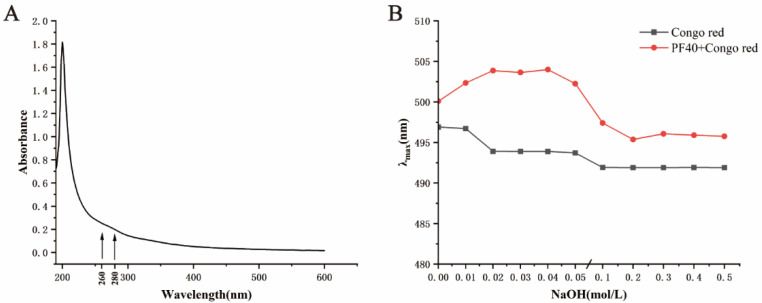
(**A**) UV spectrum and (**B**) Congo red test results of PF40.

Nucleic acids and proteins have significant UV absorption around 260 nm and 280 nm, respectively. UV analyses are often used to assess the purity of polysaccharides [22]. The UV spectrum of PF40 is presented in Figure 4A. PF40 had no absorption peak at 280 nm and 260 nm. The largest absorbance peak of PF40 was 200 nm, which showed features of polysaccharides. All of these attest to the fact that PF40 did not contain protein and nucleic impurities and, therefore, had high purity.

### 2.4. Congo Red Test

The polysaccharide with a three-helix configuration can form a compound with Congo red under a weak alkaline solution, whose maximum wavelength of absorption can be redshifted when contrasted with Congo red [23]. The three-helix configuration in the polysaccharide under study was wrecked, and the redshift phenomenon of the polysaccharide–Congo red combination was attenuated, with the intervention of strong alkali. The λmax values of the PF40–Congo red combination in NaOH solution (0–0.5 M) are presented in Figure 4. The maximum absorbance of the mixture redshifted, with an increase in the concentration of NaOH solution. The highest value of λmax was achieved when the concentration of NaOH solution reached 0.05 M. The absorption gradually decreased until it reached a constant, alongside the continued increase in concentrations. The findings of this study indicated that PF40 might have triple-helix structural conformations in an aqueous solution. Generally, polysaccharides with triple-helix structural conformations usually have higher biological activity [24,25].

### 2.5. Thermogravimetric Analysis

**Figure 5:**
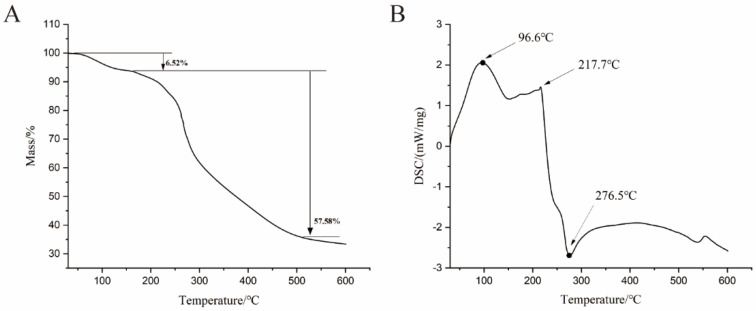
(**A**) TG and (**B**) DSC curves of PF40.

Thermal stability is an important parameter for bioactive molecules [26]. Thermogravimetric analysis (TG) of PF40 (Figure 5) was executed to investigate the thermal stability and degradation pattern of polysaccharides. PF40 showed three stages of weight loss. A slight weight loss was observed at the first stage (30–168 °C), which was caused by the loss of bound moisture from the polysaccharide. During the second phase (168–451 °C), a rapid weight loss occurred, which mainly involves the degradation of the polymer, which then leads to the production of gaseous products. Then, in the third stage (451–600 °C), weight loss occurred, which is related to the oxidation of organic matter. Differential scanning calorimetry (DSC) was further employed to determine the endothermal or exothermal condition of polysaccharides with increasing temperature. The transformation temperatures (T_0_ and Tp) were around 96.6 and 276.5 °C. The structure of PF40 began to collapse rapidly after 217.7 °C. The results from TG and DSC showed that PF40 had a relatively stable structure under 168 °C. PF40 can be used as a steadied additive in food formulations, which are subjected to high temperatures in the course of their processing [27].

### 2.6. Zeta Potential

**Figure 6:**
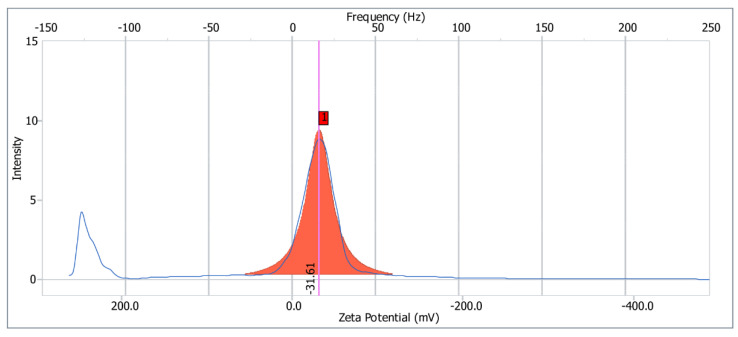
Zeta potentials of PF40.

The charges of the compound might reflect the stability of the solution, which directly determines the compound’s possible application scenarios [28]. The zeta potentials of PF40 were determined, as shown in Figure 6. PF40 was negatively charged, and the zeta potential of PF40 was −31.61 mV. These results confirm that PF40 is a non-ionic polysaccharide [29,30].

### 2.7. XRD Analysis

**Figure 7:**
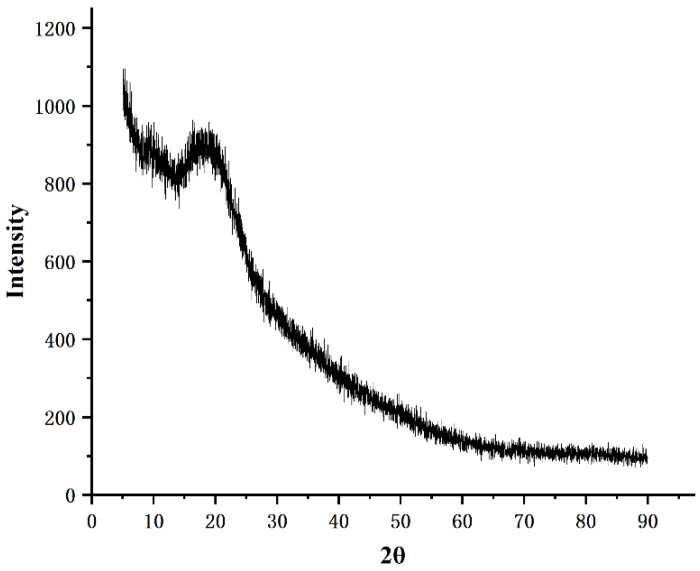
X-ray diffraction pattern of PF40.

XRD tools have been widely adopted to explore the crystalline structure of Biological Macromolecules [31,32]. For the most part, amorphous materials present as broad diffraction peaks, whereas crystalline components present as sharp peaks [33]. The X-ray diffraction pattern of PF40 is shown in Figure 7. PF40 showed a broad diffraction peak only at approximately 15° (2θ), with no sharp and strong diffraction peaks in the 2θ range from 5° to 90°. This indicates that PF40 cannot form a single crystal and mainly exists in an amorphous form [34].

### 2.8. SEM Analysis

**Figure 8:**
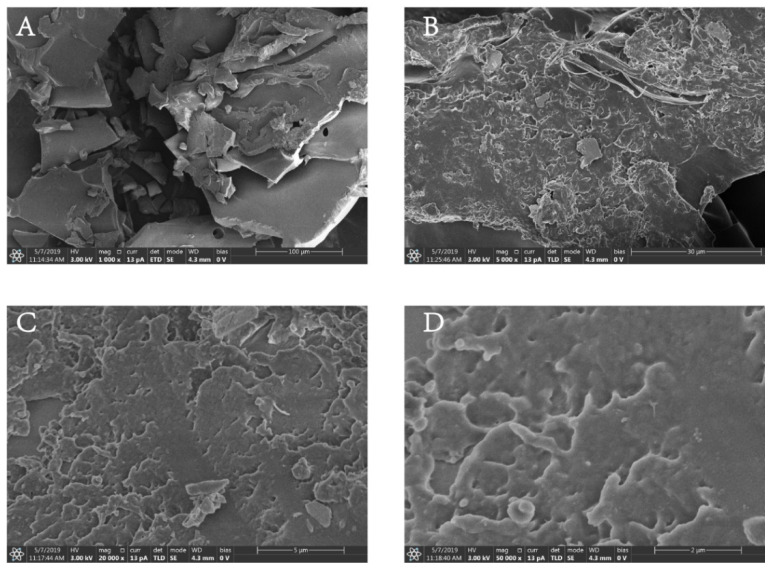
Scanning electron microscopic (SEM) images of PF40: (**A**) 1000×; (**B**) 5000×; (**C**) 20,000×; (**D**) 50,000×.

The micropattern of PF40 was explored via SEM at different amplification (1000×, 2000×, 5000×, and 10,000×). As shown in Figure 8, PF40 showed a shattered and sheet-like shape at the amplification of 1000×, 2000×. It is conjectured that this phenotype might be caused by crosslinking between sugar chains, indicating a strong interplay between the molecules of the polysaccharide. The superficial of PF40 when evaluated at the amplification of 5000× and 10,000× seemed to be slick, with some fine stripes and perforated cavities, which might be created by the sublimation of ice crystals during lyophilization or may be correlated with the branching of the polysaccharides [35]. These results indicate that PF40 has an amorphous structure, and they are consistent with the results of the XRD analysis.

### 2.9. Molecular Imaging Analysis

**Figure 9:**
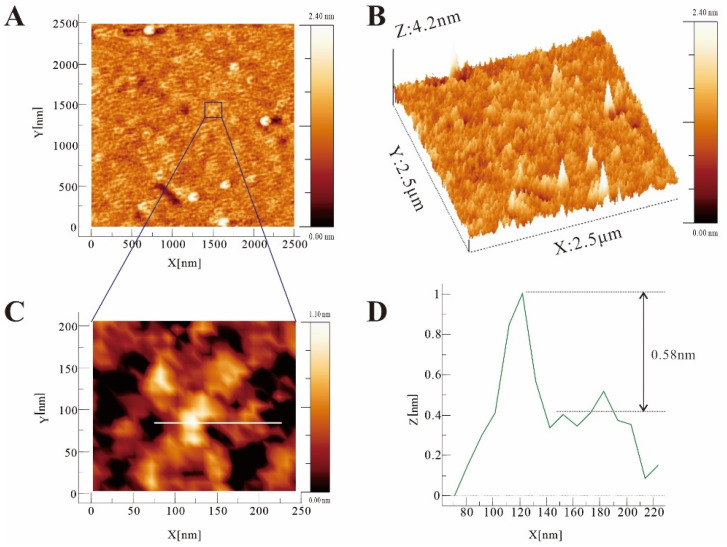
SPM images of PF40 deposited from a 1 μg/mL solution: (**A**) a 2D image of PF40, image size: 2.5 μm × 2.5 μm; (**B**) a 3D image of PF40, image size: 2.5 μm × 2.5 μm; (**C**) SPM image of a single molecule of PF40, image size: 250 nm × 200 nm; (**D**) cross-section shown by the white line in (**C**).

SPM was used to observe the nanoscale microstructure of biologically active macromolecules [36,37]. The two-dimensional and stereoscopic images of PF40 are shown in Figure 9. The surface morphology of PF40 presented irregular convex features. Furthermore, a detailed SPM image and cross-sectional height measurement of PF40 in the extremely diluted solution are shown in Figure 9C,D. The PF40 molecule exhibited a multi-branched structure, extending from the center to the periphery, with a diameter of about 150 nm. The cross-sectional height of PF40 represented by a white line is shown in Figure 9D. The height was measured to be roughly 0.58 nm. The average height of the polysaccharide chain ranges approximately from 0.1 to 1.0 nm, as previously reported [17]. Therefore, the image shown in Figure 8C might correspond to a single-molecule observation of PF40.

### 2.10. Effects of PF40 on Fasting Blood Glucose, Insulin Levels, and HOMA-IR in T2DM Rats

As seen in Table 1, compared with the normal group, the model group induced insulin resistance, characterized by hyperglycemia, hyperinsulinemia, and increased HOMA-IR. Treatment with PF40 or metformin alone reduced fasting blood glucose levels, serum insulin levels, and HOMA-IR, compared with the model group. Interestingly, the PF40 + metformin group had a greater reduction than either PF40 or metformin alone. These findings suggest that PF40 combined with metformin treatment alleviates glucose and insulin intolerance in T2DM model rats.

### 2.11. PF40 Reduced Jejunal Tissue Damage in T2DM Rats

**Figure 10:**
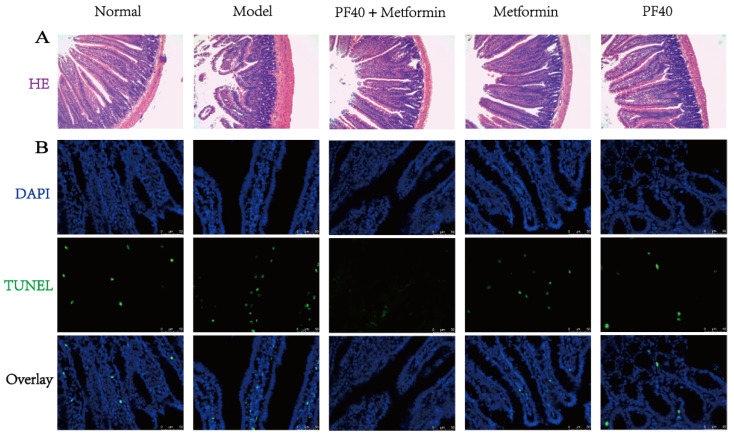
Effects of PF40 on histopathology of jejunum in T2DM rats: (**A**) HE staining, magnification ×200; (**B**) immunofluorescence images, magnification ×400, DAPI-labeled nuclei (blue fluorescence), and FITC-conjugated dUTP (green fluorescence) are indicated.

As shown in Figure 10, HE staining results indicated natural gland and epithelium structure, as well as complete goblet and crypt cells in the normal group. The model group showed strong signs of inflammation response, including the destruction of the epithelial structure, loss of recess, and an increase in the number of inflammatory cells. Interestingly, treatment with PF40 attenuated these intestinal inflammatory responses, particularly for the villus structure, which was relatively villus. To further determine the effect of PF40 on the apoptosis of jejuna mucosal cells, we used the TUNEL method to detect the apoptosis of jejuna cells and found that compared with the blank group, the number of apoptotic cells in the model group was significantly raised. However, the apoptotic number of jejuna tissue cells was significantly reduced after PF40 treatment, and it was better than the metformin group. The numbers of apoptosis in the PF40 + metformin group were the least, which was close to the blank control group. These results imply that PF40 at the histological level prevents the symptoms of intestinal inflammation and favors the maintenance of mucosal integrity in the gut.

### 2.12. PF40 Restored Th17/Treg Balance

**Figure 11:**
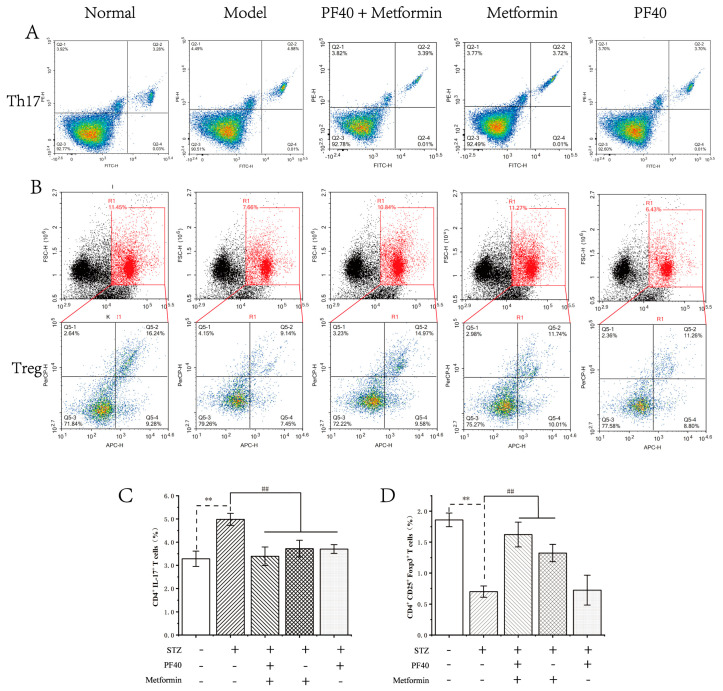
Effects of PF40 on the proportion of Th17 and Treg cells in the T2DM rats (*n* = 5): (**A**,**B**) proportion of Th17 and Treg cells from mice jejuna tissue using the flow cytometry assay; (**C**,**D**) statistics analysis of the proportion of Th17 and Treg cells. Data are presented as the M ± SD (*n* = 5 per group). ** *p* < 0.01, compared with normal group; ^##^ *p* < 0.01, compared with model group.

The balance of Th17/Treg evolution is critical for the maintenance of intestinal homeostasis [38]. To explore whether PF40 was beneficial to maintaining the balance of Th17/Treg cells, we investigated the ratios of Th17/Treg cells in jejunum tissue using the FCM technique. As depicted in Figure 11, compared with the normal group rats, a significant increase in Th17 (CD4^+^IL-17^+^) cells was identified, compared with a marked decrease in the ratios of Treg (CD4^+^CD25^+^Foxp3^+^) cells in model group rats. However, the immunological imbalance of Th17/Treg cells was markedly reversed with PF40 treatment. Importantly, the combination of PF40 and metformin had the best therapeutic effect in restoring Th17/Treg balance, compared with groups treated with PF40 or metformin alone.

### 2.13. Effects of PF40 on the Expression of Transcription-Related Proteins and mRNAs in Treg and Th17 Cells

**Figure 12:**
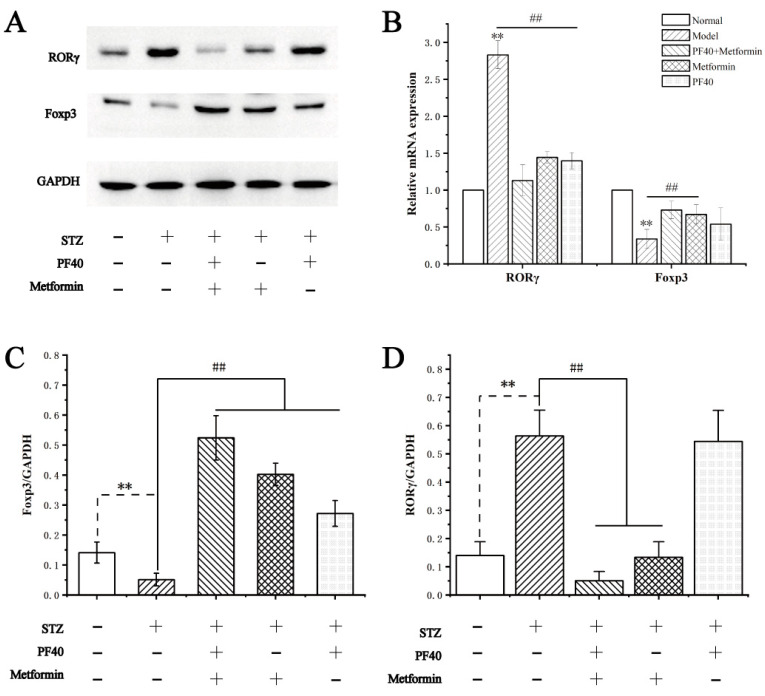
Effects of PF40 on the expression levels of Foxp3 and RORγ in T2DM rats: (**A**) the relative expression levels of proteins using WB assay; (**B**) the relative gene expression via RT-qPCR assay; (**C**) the band intensity of Foxp3 quantified with Quantity One; (**D**) the band intensity of RORγ quantified with Quantity One. Data are presented as the M ± SD (*n* = 3 per group). ** *p* < 0.01, compared with normal group; ^##^ *p* < 0.01, compared with model group.

We further investigated the molecular mechanism of restoring the Th17/Treg balance via oral administration of PF40. Previous studies proved that RORγ and Foxp3 promote differentiation regulation of Th17 and Treg cells, respectively [39,40]. The protein expressions of RORγ and Foxp3 in the jejunal were determined by using a Western blot assay. As shown in Figure 12A,C,D, the protein levels of RORγ in the model group were higher than those in the control group (*p* < 0.01). In comparison, Foxp3 protein expression showed the opposite result. After PF40 + metformin administration, the levels of RORγ were remarkably inhibited (*p* < 0.01), and Foxp3 was enlarged (*p* < 0.01), compared with the model group. As shown in Figure 12B, we further verified the expression of the above three proteins by real-time quantitative PCR method and obtained similar results. These results indicate that PF40 combined with metformin treatment exhibit stronger pharmacological activity on the expression of Foxp3 and RORγ.

### 2.14. Effect of PF40 on the Levels of Cytokines

**Figure 13:**
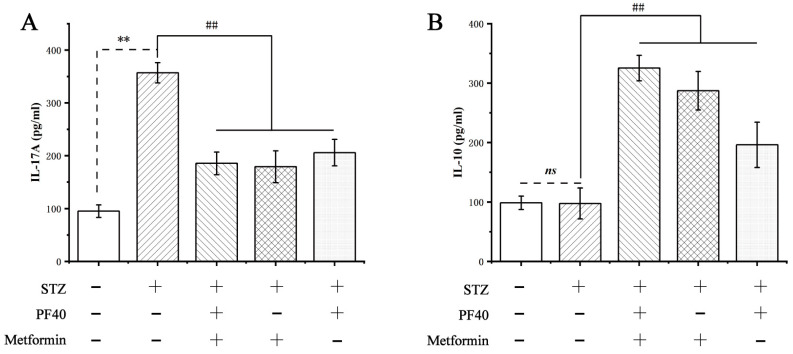
Effects of PF40 on the levels of (**A**) IL-17A and (**B**) IL-10 in T2DM rats. Data are presented as the M ± SD (*n* = 9 per group). ** *p* < 0.01, *ns*, non-significant difference, compared with normal group; ^##^ *p* < 0.01, compared with model group.

To further validate the positive effect of PF40 on Th17 and Treg cell balance, we determined the levels IL-10 of and IL-17A, which are specifically secreted in Treg cells and Th17 cells [41]. As shown in Figure 13, the levels of IL-17A in the model group were appreciably increased, compared with those of the control group (*p* < 0.01). In contrast to the model group, after treatment with PF40 or metformin, the levels of IL-17A in the serum of T2DM rats were significantly reduced (*p* < 0.01), and IL-10 levels were increased significantly (*p* < 0.01). These results suggest that PF40 may be beneficial to restore the STZ-induced imbalance of Th17 and Treg cells, thereby maintaining immunoinflammatory homeostasis.

## 3. Materials and Methods

### 3.1. Materials

*Pseudostellaria heterophylla* samples were from Zherong County, Fujian Province of China. Ethanol, phenol, sulfuric acid, D_2_O, and Congo red were bought from Sinopharm (Shanghai, China). Metformin was produced by Merck Serono (Genf, Switzerland). Streptozocin (STZ) was purchased from Sigma-Aldrich (St. Louis, MO, USA). Interleukin-17A (IL-17A), ELISA kits for insulin, and interleukin-10 (IL-10) were purchased from Multi Sciences (Hangzhou, China). TB Green™ Premix Ex Taq™ II and advantage RT-for-PCR Kit were purchased from TaKaRa (Tyoto, Japan). Rat anti-CD4-FITC, rat anti-17A-PE, rat anti-CD25-APC, and rat anti-Foxp3-PerCP were from Thermo Fisher (Waltham, MA, USA). All other reagents used in this study were provided by commercial suppliers without providing any treatments unless noted otherwise.

### 3.2. Preparation and Validation of PF40

The dried TZS medicinal materials were pulverized and sieved through a 40-mesh sieve. The TZS polysaccharide (PF40) was prepared via a previously reported method, and then the composition of PF40 was verified [13]. The result indicated the monosaccharide composition was galacturonic acid (8.21%), glucose (85.97%), galactose (2.59%), and arabinose (3.23%). The molecular weight of PF40 was 52–210 kDa.

### 3.3. FT-IR Spectroscopy

FT-IR spectrum was used to analyze the chemical bond and functional groups of PF40 [42]. KBr powder (spectroscopic grade) was used to mix with the PF40 powder samples (5 mg), and then the mixed powder was pressed into a 1 mm pellet for FT-IR mensuration. A Fourier transform infrared spectrophotometer (AVATAR 360, Nicolet, Dracut, MA, USA) was adopted to record the FT-IR spectrum with a frequency ranging from 400 to 4000 cm^−1^.

### 3.4. NMR Analysis

The structural characteristics of PF40 were analyzed via NMR spectra [43]. PF40 solution (50 mg/mL) was prepared with D_2_O as solvent. The PF40 solution was transferred to an NMR tube, and the 13C NMR and 1H NMR spectra were listed using Bruker Avance Ⅲ 400.

### 3.5. UV Spectroscopy Analysis

The MilliQ water was used to dissolve the PF40 sample to prepare a solution (2 mg/mL). The absorbance was recorded by ultraviolet spectrophotometer with a wavelength between 190 and 600 nm. Whether PF40 contains nucleic acid and protein was assessed by observing if absorption peaks at 260 and 280 nm wavelengths were noted.

### 3.6. Congo Red Experiment

Congo red test of PF40 was conducted based on a previously reported method [44], after slight modifications. Briefly, we mixed 2 mL PF40 solution (1 mg/mL) with 2 mL Congo red solution (150 μM). Various concentrations of NaOH solution, with the eventual concentrations of NaOH being 0, 0.01, 0.02, 0.03, 0.04, 0.05, 0.1, 0.2, 0.3, 0.4, and 0.5 M, respectively, were gradually added to the mixture. After incubation (10 min, 25 °C), the maximum absorption wavelength (λmax) was recorded at 400–600 nm. MilliQ water was used as a blank control group. To draw the experimental results, the concentration of NaOH (mol/L) and λmax were taken as horizontal and vertical coordinates.

### 3.7. Thermal Analysis

Differential scanning calorimetry was used to study the thermal behavior of PF40 [45]. A thermogravimetric analyzer (DSC214, Netzsch, Selb, Germany) was used for the TG and DSC analysis of the lyophilized PF40 powder. Briefly, 2.0 mg PF40 were laid in an Al_2_O_3_ crucible and heated from 30 °C to 600 °C under a protective nitrogen atmosphere (20 mL/min). Calorimetry calibration was conducted prior to determination.

### 3.8. Determination of Surface Charge

According to the method described by Jayaram Kumar [46], the mean values of measured zeta potentials were calculated to identify the surface charge of PF40. MilliQ water was used to prepare the stock solutions of PF40 and heated at 80 °C for 30 min. The solutions were made up to 0.1% concentration with MilliQ water. Finally, a zeta potential and nanoparticle analyzer (NanoPlus3, Micromeritics, Norcross, GA, USA) was used to determine the zeta potential of PF40.

### 3.9. XRD Analysis

An X-ray diffractometer (Empyrean, PANalytical B.V.) was used to determine the crystal characteristics of PF40 [47]. First, a 10 mg polysaccharide sample was ground into powder for determination. The test conditions were as follows: (1) Cu target was obtained by taking Kα as a radiation source; (2) 40 KV of voltage, 30 mA of current, and angular range of 5–90° (2θ).

### 3.10. Scanning Electron Microscopy Analysis

To obtain visual evidence of the shape and surface characteristics of polysaccharides, SEM analysis was used in this study to examine the surface characteristics of PF40 via a scanning electron microscope system (Verios G4, FEI, Waltham, MA, USA) [48,49]. The PF40 sample was sputtered with gold using a sputter coater (208 HR, Cressington, Watford, UK). Images were taken under different magnifications (10,000×, 5000×, 2000×, and 1000×).

### 3.11. Molecular Imaging Analysis

The nanoscale microstructure of PF40 was observed via scanning probe microscopy [37,50]. PF40 powder was prepared into a solution of 1 mg/mL with MilliQ water and heated at 80 °C for 20 min to make it fully dissolved, and then diluted to 1 μg/mL with MilliQ water, after which 0.5 μL was added onto fresh mica and dried at room temperature. SPM images were carried out using a Dimension Icon scanning probe microscope (Bruker, Karlsruhe, Germany) and obtained with tapping mode.

### 3.12. Animal Welfare and Ethical Statements

Sixty male Sprague Dawley (SD) rats (4 weeks, 180–200 g, SPF) were obtained from Shanghai Slaccas Company, with certificate No. SCXK (Shanghai, China) 2017–0005. All animals were housed in a standardized laboratory animal housing room. All procedures and protocols were approved by the Institutional Animal Care and Use Committee at the Fujian Academy of Chinese Medical Sciences (no. FJATCM-IAEC2020002). All experiments were conducted by following the “Guide for the Care and Use of Laboratory Animals” developed by the National Academy of Science and released by the National Institute of Health.

### 3.13. Establishment of an Insulin-Resistant T2DM Rat Model

Ten rats were randomly allocated as control rats, which were ingested an ordinary diet including 25% flour, 25% cornmeal, 25% wheat, 8% fish flour, 10% bean powder, 2% yeast, 4% bone meal, and 1% refined salt. Other rats were fed a high-fat–sugar diet (including 10% casein, 20% sucrose, 15% Lard, 0.6% calcium hydrophosphate, 0.2% sodium cholate, 1.3% cholesterol, 0.4% stone powder, 0.4% premix, and 52.2% basic material) for 3 weeks. By the end of week 3, all rats fasted overnight but could drink water freely. All rats were intraperitoneally (*i.p.*) injected with the freshly prepared STZ solution (0.021 mol/L pH 4.5 sodium citrate buffer, 0.5 mL/100g bw) to induce diabetes mellitus. Three days after the STZ injection, rats were considered to be T2DM rats if fasting blood glucose concentrations were 16.7 mmol/L or above. Thirty-eight rats were successfully induced with diabetes. The T2DM rats were separated into 4 groups as follows: diabetes model group (model, *n* = 10); PF40 group (PF40, 1.5 g/Kg bw, *n* = 10); metformin group (metformin, 135 mg/kg bw, *n* = 9); and PF40 combined with metformin group (PF40 + metformin, PF40, 1.5 g/Kg bw; metformin, 135 mg/kg bw, *n* = 9). Except for the rats in the control group, all rats were fed a high-fat–sugar diet daily for 4 weeks via intragastric administration.

### 3.14. Biochemical Index Tests

At week 4, the rats fasted for 8 h, and their blood samples were gathered from the tail vein. The level of blood glucose was gauged using a blood glucose meter (HEA-214, OMRON, Dalian, China) according to the manufacturer’s instructions. The rats fasted for 12 h prior to the last administration, and blood was subsequently collected from the abdominal aorta and then centrifuged (1200 g, 10 min) to separate the serum before being saved at −80 °C until analysis. An ELISA kit was utilized to detect the fasting serum insulin levels. Insulin resistance was assessed with HOMA-IR. The calculation formula of HOMA-IR: glucose level (mmol/L) × insulin levels (mU/mL)/22.5. Cytokine (IL-17A and IL-10) levels of serum samples were evaluated via ELISA kits according to the manual provided by the manufacturer.

### 3.15. Morphological Analysis

The jejunum tissues were fixed using formaldehyde solution (4%) (Beyotime, Shanghai, China), dehydrated, embedded into wax blocks, and cut into paraffin sections. The morphological changes were assessed under a light microscope after hematoxylin-and-eosin (H&E) staining. The TUNEL assay was used to determine cell apoptosis in jejunum tissues [51].

### 3.16. Flow Cytometry Analysis

The ratio of Th17 and Treg cells in jejunum tissue was determined with FCM [52]. Jejunum tissue was lysed into single-cell solutions with DNase I (0.1 mg/mL) and collagenase type D (1 mg/mL) in Hanks’ Solution (30 min, 37 °C). The red cells were lysed using red blood cell lysis buffer. The cells were washed 3 times with Hanks’ solution for FCM analysis. To identify Th17 cells, we combined plated cells with ionomycin (1 ng/mL), along with PMA (5 ng/mL) for 5 h, and brefeldin A (10 ng/mL) was added after 30 min. Cells were washed with Hanks’ Solution and stained using 5 µL anti-CD4-FITC, before proceeding to permeabilization using cytofix/cytoperm, after which 5 µL of anti-IL-17A-PE was added. For detecting Treg cells, a similar pretreatment protocol was applied; however, cells were stained using a 5 µL anti-Foxp3-PE. After washing, cells were further stained with anti-CD25-APC and anti-CD4-FITC. For the last step, cells were rinsed with a buffer solution, and analysis was performed in an FCM buffer. The percentage of CD4^+^CD25^+^Foxp3^+^ T cells and CD4^+^IL-17^+^ T cells was determined using NovoCyte flow cytometers (ACEA, San Diego, CA, USA). Data collection and analysis were from three independent samples.

### 3.17. Western Blot Analysis

The protein samples from each of the groups were analyzed using SDS–PAGE. A RIPA buffer containing protease and phosphatase inhibitor cocktail was utilized to homogenize the jejunum tissues. A BCA Protein Assay Reagent Kit (Pierce™ BCA, Thermo Scientific, Waltham, MA, USA) was utilized to identify the protein concentrations of tissue homogenates. The tissue homogenates with equal amounts of protein (30 μg) were subsequently subjected to SDS–PAGE and transferred to PVDF membranes. The PVDF was incubated with rabbit monoclonal primary antibodies against Foxp3, RORγ, and GAPDH, respectively, following a blockage using 5% non-fat dry milk in PBS. After washing, PVDF was mixed with horseradish-peroxidase-conjugated goat anti-rabbit IgG and visualized via ECL Western Blotting Detection Reagent (Pierce™ Fast Western, Thermo Scientific, Waltham, MA, USA). Protein bands were identified using the ChemiDoc XRS Image System (ChemiDoc XRS, Bio-Rad, Hercules, CA, USA), and the band density was further measured using Quantity One software and evaluated as a percentage of GAPDH [53].

### 3.18. Real-Time Quantitative PCR Analysis

Total RNA of jejunum tissue from different groups was extracted using Trizol reagent and spectrophotometrically quantified at OD_260_/OD_280_. The obtained RNAs were reverse transcribed to cDNA using Advantage RT-for-PCR Kit (TaKaRa, Tyoto, Japan). The levels of expression for the target genes were evaluated with real-time quantitative PCR using TB Green™ Premix Ex Taq™ II (TaKaRa, Tyoto, Japan). Relative mRNA expression levels of the intention genes were computed via a 2^−∆∆CT^ method, normalized to the internal control using GAPDH.

### 3.19. Statistical Analysis

SPSS 22.0 was employed to analyze the data. Mean and standard deviation (SD) were used to present the data. Basic data were analyzed using a *t*-test (two-group comparisons) or one-way ANOVA (three or more groups of comparisons). *p* < 0.05 was used to indicate the significant difference.

## 4. Conclusions

The ever-rising attention in the search for new food additives and pharmaceutical preparations from nature’s treasures to replenish chemistry medicine is a growing tendency, as the latter applied alone has been proved to have a variety of adverse reactions. Polysaccharides are considered one of the promising candidates because they have many biological functions. Our previous studies demonstrated that *Pseudostellaria* polysaccharides could reduce fasting blood glucose and improve insulin resistance in T2DM rats. However, the physicochemical properties and antidiabetic mechanisms of PF40 were unclear. In this study, we showed the physicochemical properties of PF40 and explored the possible mechanism of antidiabetic activity. The monosaccharide residue of PF40 has an α-pyranoid ring. PF40 mainly exists in amorphous form with a triple-helix conformation. The single-molecular structure of PF40 exhibits a multi-branched structure extending from the center to the periphery. PF40 is a non-ionic polysaccharide with a flaky and smooth surface morphology and exhibits good thermal stability below 168 °C. Experimental studies on antidiabetic characteristics found that PF40 can significantly improve STZ-induced intestinal mucosal damage and reduce the apoptosis of villus epithelial cells. PF40 combined with metformin significantly improved insulin resistance in T2DM rats. After combined treatment, the expression of RORγ protein in jejunum tissue of T2DM rats declined significantly, the expression of Foxp3 protein was markedly increased, and the STZ-induced Th17/Treg cell imbalance was inhibited. Interestingly, the effects of PF40 combined with metformin were better than those observed by using PF40 or metformin treatment alone. Therefore, the present study suggests that the antidiabetic properties of PF40 in treating T2DM may be through inhibiting the expression of RORγ protein and increasing Foxp3 protein in the jejunum of T2DM rats, and then restoring the STZ-induced imbalance of Th17/ Treg cells, thereby maintaining intestinal immune homeostasis. These results imply that PF40 from *Pseudostellaria heterophylla* can be used in functional foods or as antidiabetic agents.

## Figures and Tables

**Table 1:** Effect of PF40 on fasting blood glucose, insulin levels, and HOMA-IR in T2DM rats (*n* = 9).

	Blood Glucose (mmol/L)	Insulin Levels (mU/L)	HOMA-IR
Normal	5.14 ± 0.89	8.58 ± 0.24	1.95 ± 0.33
Model	17.22 ± 7.48 **	12.17 ± 0.23 **	10.90 ± 2.00 *
PF40 + metformin	6.52 ± 2.21 ^##^	8.73 ± 0.38 ^##^	2.55 ± 0.99 ^##^
Metformin	8.86 ± 3.81 ^#^	8.14 ± 0.38 ^##^	3.19 ± 1.34 ^##^
PF40	8.90 ± 5.61 ^#^	9.12 ± 0.7 ^##^	3.63 ± 2.41 ^##^

Compared with normal group: * *p* < 0.05, ** *p* < 0.01; compared with model group: ^#^ *p* < 0.05, ^##^ *p* < 0.01.

## Data Availability

The data presented in this study are available on request from the corresponding author.

## Sample Availability

Samples of the compounds are available from the authors.
